# ‘Sclérose du col vésical’: An obsolete terminology still used by French literature?

**DOI:** 10.1080/2090598X.2022.2092994

**Published:** 2022-07-01

**Authors:** Jihad El Anzaoui, Chatar Achraf, Akajai Ali, Amaziane Ahmed, Lakrabti Naceur, Habyebete Soufiane, Abdelghani Ammani

**Affiliations:** 1 Head of urology department, Military Hospital My Ismail, 50000, Meknes University Sidi Mohamed Ben Abdellah, Faculty of medicine and pharmacy in Fes, Morocco jihad.elanzaoui@usmba.ac.ma; 2 Urology Department, Military Hospital My Ismail Meknes, Morocco; 3 Urology department, Military Hospital My Ismail, University Sidi Mohamed Ben Abdellah, Faculty of medicine and pharmacy in Fes, Morocco

Dear Sir,

The evolution of medicine is taken place towards the homogenization of medical terms.

**Figure 1.**
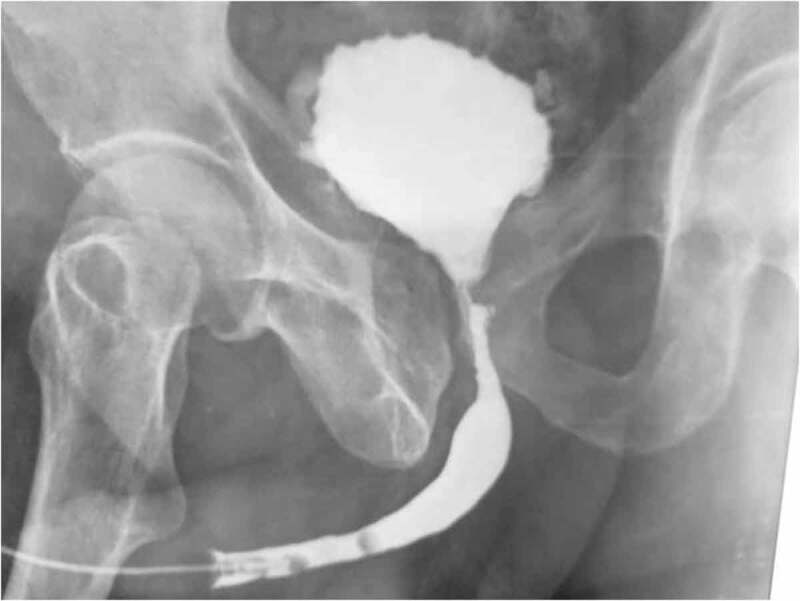
Retrograde opacification of the urethra showing a bladder neck stenosis post transurethral resection of the prostate for prostate adenoma.

The bladder neck contracture (BNC) is a well-known condition to urologists, described as a fibrous narrowing of the bladder neck more or less extended to the prostatic and posterior urethra (Figure 1). It usually occurs as a complication of a surgical or radiotherapeutic approach to the prostate.

Albeit its diagnostic and therapeutic aspects have been extensively studied in the literature, its terminology remains a subject of ambiguity, especially in French literature.

The French term of « sclérose du col vesical » or « sclérose de la loge prostatique » refers to this entity. In contrast to English literature, which mainly uses the term ‘bladder neck contracture’ or ‘stenosis’ rather than ‘bladder neck sclerosis’, the term ‘sclérose du col vésical’ is the principal terminology used in French literature.

In fact, the International Society of Urology (SIU) and the International Consultation on Urological Diseases (ICUD) published, in 2014, recommendations regarding the accuracy of urethral terminology [1].

According to these recommendations, the term ‘bladder neck sclerosis’ should be replaced by ‘bladder neck stenosis’ or ‘stenosis of the vesico-urethral anastomosis’.

Despite the effort of learned societies to universally homogenize the medical language, many urologists do not adhere to this terminology and prefer old terms in common practice. This lack of adherence can be seen in both English and French literature.

A review of the literature on PubMed, Web of Science, Scopus, and Google Scholar of articles published from 2015 to 2021 using the French terms (sclérose du col), (sclérose de l’anastomose vésico-uréthrale), or (sclérose de la loge prostatique) found 16 articles published in French that continue to use the term ‘sclérose’ to designate the said condition (Table 1), which proves the wide persistent use of this terminology.

**Table 1.** List of French articles that have used the term ‘sclérose du col vésical’ between 2015 and 2021.

	Authors, year	Journal	Title
1	Comat et al. 2015	progrès en urologie	Énucléation de la prostate au laser Holmium (HoLEP): expérience monocentrique après 800 procédures.
2	L. salomon et al. 2015	progrès en urologie	Résultats fonctionnels et prise en charge des troubles fonctionnels après prostatectomie totale.
3	V. Misrai et al. 2015	progrès en urologie	Complications graves et inattendues de la chirurgie de l’hyperplasie bénigne de prostate: résultats de l’enquête du CTMH auprès des urologues de l’AFU
4	R. Thuret et al. 2016	progrès en urologie	- Troubles du bas appareil urinaire et de la statique pelvienne chez les candidats et receveurs d’une transplantation rénale
5	J.L. hoepffner et al. 2016	bulletin de cancer	Prostatectomie radicale mini-invasive: apport de l’assistance robotisée, résultats fonctionnels et oncologiques.
6	M. Mouton et al. 2017	progrès en urologie	Énucléation prostatique au laser holmium (HoLEP): retour sur 5 ans d’expérience et 1201 cas.
7	M. thoulouzan et al. 2017	progrès en urologie	Résultats de la photovaporisation prostatique par laser Green Light XPS-180 W dans l’hyperplasie bénigne de prostate (HBP) de gros volume (≥ 80 mL).
8	J. calves et al. 2017	progrès en urologie	.Résultats fonctionnels à long terme après photo-vaporisation laser prostatique au Greenlight XPS
9	A. de la Taile et al. 2018	progrès en urologie	Conséquences de l’obstruction prostatique sur le fonctionnement vésical, impact de la désobstruction, et prise en charge des récidives après chirurgie
10	G. Robert et al. 2018	progrès en urologie	Traitements chirurgicaux de l’obstruction prostatique bénigne: standards et innovations
11	A. descazeaud et al. 2018	progrès en urologie	Conséquences sexuelles des traitements de l’HBP
12	A Descazeaud et al. 2018	progrès en urologie	Prise en charge de l’obstacle sous vésical lié à une HBP chez les patients à terrain particulier et/ou ayant une complication
13	M. gaullier et al. 2020	progrès en urologie	Sclérose du col: lambeau d’avancement de muqueuse vésicale par voie coelioscopique robot assistée.
14	M. El Akri et al. 2020	progrès en urologie	Traitement d’une sclérose complète du col vésical post-prostatectomie par réfection de l’anastomose urétro-vésicale par voie périnéale.
15	L. Freton et al. 2020	progrès en urologie	Plastie yv et plastie postérieur robotique pour sclérose de col vésical post-pvp.
16	S. Bart et al. 2020	progrès en urologie-FMC	Chirurgie et règles de l’art.

This letter aims to draw the attention of authors and reviewers to the fact that this terminology is misleading and was previously revisited by learned societies.

The term ‘sclerosis’ derives from the Greek word ‘sklēroun’ meaning harden.

The French dictionary of ‘Académie de Medecine’ defines ‘sclérose’ as a pathological induration of a tissue affected by fibrosis [2].

The addition of the character of hardness is not constant in all dictionaries. The French dictionary ‘Larousse médical’, for example, considers fibrosis as equivalent to sclerosis [3].

Considering sclerosis as equivalent to fibrosis or just a type of it, Kaynar et al. in 2016, by analyzing the resected specimens of 338 cases of BNC, found varying degrees of inflammation and fibrosis [4].

For all the examined specimens, the term ‘sclerosis’ was not used by the anatomopathologists.

Moreover, fibrosis is a physiological healing process constantly found in all previously injured tissues, either by trauma, instrumental maneuvers, infections, or inflammatory processes.

In fact, any normal tissue healing is a stepwise process starting with hemostasis followed by inflammation, proliferation, and ultimately regenerating and remodeling.

During the inflammatory phase, immunocompetent cells are activated to release cytokines, which induce matrix deposition, fibrosis, additional cell proliferation, and angiogenesis.

Applied to the prostate, either in canine model or in human studies, the injury to the prostate by monopolar resection, bipolar resection, or laser exposition triggers an immediate phase of burning necrosis and hemorrhage. The subsequent delayed phases consist of a mild chronic inflammatory infiltration, fibrosis, and squamous metaplasia, gradually replaced by urothelial re-epithelialization [5,6].

On the molecular level, the reasons for the worse evolution to BNC, in some cases, remain to be elucidated. The identification of some clinical risk factors does not explain the primum movens triggering the disorder or why some patients progress to stricture despite a well-conducted prostate treatment [7].

Likewise, the term ‘sclerosis’ is still widely used in other fields of medicine, describing many medical conditions characterized by tissue alteration: scleroderma (sclérodermie), multiple sclerosis (sclérose en plaque), peritoneal sclerosis (sclérose péritoneale), etc.

Guido et al., in 2006, discussed the accuracy of the term sclerosis in the description of a condition called ‘peritoneal sclerosis’. The authors emphasize the discrepancy between peritoneal sclerosis described as a late and irreversible end stage of inflammation, and another entity described as ‘simple peritoneal fibrosis’, more benign and premature [8].

Another example is the case of nephrosclerosis. Glomerulosclerosis is considered a late evolution of a long process of fibrosis [9].

At the urethral level, spongio-urethral fibrosis was divided into three categories of increasing severity [10].

Teresa Olsen et al. described spongio-urethral sclerosis as a grade III referring to the most aggressive and advanced stage of fibrosis.

Ultimately, it seems that the nosological confusion between fibrosis and sclerosis, from a linguistic point of view, is based on pathophysiological and histological confusions.

BNC is a hypertrophic scar of the bladder neck and prostate cavity adequately described as a ‘stenosis’ or narrowing. ‘Stenosis’ describes the etiological factor from which originate all clinical and therapeutic features of the disease.

The bladder neck stenosis is an obstructive uropathy, characterized by a difficulty in micturition, which can lead to urine infection, bladder wall thickening, lithogenesis, and vesico-ureteral reflux.

The treatment of bladder neck stenosis consists in recanalization, like for any other narrowed urethral segment, using reconstructive surgery tools such incisions, plasty, and grafting.

In conclusion, the non-adhesion to international recommendations and the use of confusing terms lead to misunderstanding and lack of accuracy, which mainly alters communication and research.

Regards.
